# Trends of Antidiabetic and Cardiovascular Diseases Medication Prescriptions in Type 2 Diabetes between 2005 and 2017—A German Longitudinal Study Based on Claims Data

**DOI:** 10.3390/ijerph20054491

**Published:** 2023-03-03

**Authors:** Batoul Safieddine, Florian Trachte, Stefanie Sperlich, Jelena Epping, Karin Lange, Siegfried Geyer

**Affiliations:** 1 Medical Sociology Unit, Hannover Medical School, Carl-Neuberg-Str. 1, 30625 Hannover, Germany; 2 Accident and Emergency Department, St. Vincenz Hospital, Am Busdorf 2, 33098 Paderborn, Germany; 3 Medical Psychology Unit, Hannover Medical School, Carl-Neuberg-Str. 1, 30625 Hannover, Germany

**Keywords:** type 2 diabetes, medication prescriptions, morbidity expansion, claims data, Germany

## Abstract

Background: With an attempt to understand possible mechanisms behind the severity-dependent development of type 2 diabetes (T2D) comorbidities, this study examines the trends of antidiabetic and cardiovascular diseases (CVD) medication prescriptions in individuals with T2D. Methods: The study is based on claims data from a statutory health insurance provider in Lower Saxony, Germany. The period prevalence of antidiabetic and CVD medication prescriptions was examined for the periods 2005–2007, 2010–2012, and 2015–2017 in 240,241, 295,868, and 308,134 individuals with T2D, respectively. (Ordered) logistic regression analyses were applied to examine the effect of time period on the number and prevalence of prescribed medications. Analyses were stratified by gender and three age groups. Results: The number of prescribed medications per person has increased significantly for all examined subgroups. For the two younger age groups, insulin prescriptions decreased but those of non-insulin medications increased, while both increased significantly over time for the age group of 65+ years. Except for glycosides and antiarrhythmic medications, the predicted probabilities for CVD medications increased over the examined periods, with lipid-lowering agents demonstrating the highest increase. Conclusions: Results point towards an increase in medication prescriptions in T2D, which is in line with the evidence of the increase in most comorbidities indicating morbidity expansion. The increase in CVD medication prescriptions, especially lipid-lowering agents, could explain the specific development of severe and less severe T2D comorbidities observed in this population.

## 1. Introduction

Temporal change in morbidity has been of a high concern due to its solid effects on health policy planning and public health programming [1]. While non-communicable diseases have become the leading cause of premature morbidity globally [2], type 2 diabetes (T2D) has reached alarming levels due to its increasing prevalence and associated quality of life impairment [3].

In Germany, research suggests that morbidity in the context of T2D is expanding. As well as the fact that prevalence rates of T2D have been increasing in Germany [4], the age at onset has been shown to be declining among younger individuals [5]. On the other hand, life expectancy for individuals with T2D has been progressively increasing between the years 2005–2014 [4], indicating more years lived with the disease. Adding to that, the extra years lived with T2D are associated with more comorbidities [6]. Based on a large population of a health insurance provider in the state of Lower Saxony, Germany, our previous research has examined the development of comorbidities in individuals with T2D between the years 2005 and 2017. It indicated that individuals with T2D have significantly elevated risks of having more comorbidities diagnosed in the time period 2015–2017 compared to 2005–2007 [7]. Moreover, our study showed that the prevalence of severe adverse cardiovascular (CVD) events, such as myocardial infarction (MI) and stroke, has either remained constant for some of the age and gender groups examined, or slightly decreased over time for the other age and gender groups. However, at the same time, a clear and substantial increase was observed in other CVD comorbidities that are counted as risk factors, such as hypertension, cardiac insufficiency, and hyperlipidemia among the men and women of all age groups examined [7]. The study also reported a significant increase in the risk of having other vascular diseases, such as retinopathy, nephropathy, and polyneuropathy. Accordingly, it was concluded that the extra years lived with T2D are spent with more comorbidities, which signifies a deterioration in the quality of life and, thus, indicates an expansion of morbidity in the population of individuals with T2D.

However, the mechanisms behind the different development patterns of CVD comorbidities in individuals with T2D depending on severity remains unclear. It can be hypothesized that as a result of new treatment guidelines [8], different medication prescription practices have been developing, leading to the postponement of severe adverse health events. At the same time, deterioration of lifestyle risk factors [9] might lead to an increase in the prevalence of other chronic comorbidities, despite better treatment and diagnoses. This study will focus on the first premise of the abovementioned hypothesis through exploring temporal trends of medication prescriptions in T2D.

Studies from Europe reported evidence on an increase in the number of medications prescribed per patient [10] and polypharmacy [11] during the last two decades. Nevertheless, while studies examining time trends of antidiabetic medication use in Germany exist [12,13,14], evidence on the time trends of specific medication groups aimed at managing diabetes as well as the comorbidities accompanying it is scarce. Moreover, considering gender and age differences in the management of T2D is essential for understanding mechanisms that lie behind the morbidity development patterns in specific subgroups.

Based on the same data used in our previous research to examine the development of comorbidities in T2D [7], this study aims to explore possible mechanisms behind the severity-dependent developmental trends of T2D comorbidities through examining the gender- and age-stratified development of medication prescriptions in T2D. We hypothesize that individuals with T2D have been more routinely medicinally treated over time between the years 2005 and 2017, leading to the delay of severe CVD events. In order to examine this hypothesis, the following research questions will be addressed:

1. How has the number of prescribed medications per person been developing between the years 2005 and 2017 in individuals with T2D?
2. How has the prevalence of antidiabetic and CVD medication prescriptions been developing between the years 2005 and 2017 in individuals with T2D?

## 2. Materials and Methods

### 2.1. Data

The database for this study is anonymized claims data of individuals insured by “AOKN: Allgemeine Ortskrankenkasse Niedersachsen” that cover the years 2005–2017. AOKN is a large statutory health insurance provider in the state of Lower Saxony, Germany which insures around one-third of the population in this state [15]. Given that health insurance is mandatory in Germany, about 90% of the population are statutory insured, with insurance premiums defined individually based on income [16]. All individuals in the statutory health insurance system receive the same health care coverage. The datasets include demographic information, in- and outpatient diagnoses, medical prescriptions and medical treatments. The data are currently available for the years from 2005 to 2017, allowing for a longitudinal analysis of diagnoses and prescribed treatments in this time period. The scientific use of the pre-existing anonymized claims datasets is regulated by German law in the German Civil Code “Bürgerliches Gesetzbuch”.The data protection officer of the Local Statutory Health Insurance of Lower Saxony-AOK Niedersachsen where the headquarter is located in Hannover, Germany) has given permission to use them for scientific purposes. Therefore, no ethical approval was required for this study.

### 2.2. Definition of T2D Cases and Medications

The population of this study includes all insured individuals with T2D aged 18 years and older. T2D is defined based on individual diagnosis data, based on the German version of the International Classification of Diseases (ICD-10 GM) and on medication data. The exact definition and plausibility mechanism of this definition have been described in an earlier publication [7].

Medication groups were identified according to the anatomical therapeutic chemical classification (ATC) with daily doses defined for the German pharmaceutical market [17].

After consulting clinicians, 12 discrete diabetes and CVD medication groups were first identified, namely insulin, non-insulin, blood thinning medications, vasodilators, diuretics, beta blockers, calcium channel blockers, renin–angiotensin agents, glycosides, antiarrhythmic and antiadrenergic agents. The corresponding ATC codes of the 12 medication groups are presented in the Supplementary Materials in Table S1. Then, the discrete CVD medication groups were simplified into four major medication groups to be used in the analyses: (1) antihypertensive agents (beta blockers, calcium channel blockers, diuretics, renin–angiotensin agents, vasodilators, and antiadrenergic agents), (2) lipid-lowering agents, (3) blood thinning medications, and (4) glycosides and antiarrhythmic medications.

In order to avoid overestimation of prescription prevalence, prescriptions were only considered plausible if they were present for individuals at least twice in each time period examined, with the exception for individuals who were insured for only one quarter in the corresponding period.

### 2.3. Time Period

In this study, the trend of medication prescriptions was examined over three time periods between 2005 and 2017, which are the years for which the data are currently available, with equal intervals and gaps in-between. The three time periods were 2005–2007 (p1), 2010–2012 (p2), and 2015–2017 (p3). In order to limit bias, T2D, as well as all medication prescriptions, were newly defined in each period using the same criteria, allowing for the same potential errors and, thus, improving comparability among the time periods. The time periods approach was used in order to better illustrate clear directions of temporal development. Two-year gaps were left between the three time periods to provide sufficient time for possible changes in morbidity and prescription frequency to happen.

### 2.4. Statistical Analysis

In order to detect age and gender differences in the trends of medication prescriptions in individuals with T2D, all analyses in this study were applied separately for men and women, and for three age groups: 18–45 years, 46–64 years, and 65+ years.

Period prevalence rates of the single medication groups in the three examined time periods were calculated for all subgroups and are displayed in the Supplementary Materials in Table S2. Denominators are based on the aggregated insurance duration in each period in terms of person-years to correct for censoring that might result due to different observation periods.

#### 2.4.1. Trend of the Number of Prescribed Medications

The number of prescribed discrete medication groups (that could range between 0 and 12) was grouped into the following categories: “0 Medications”, “1–2 Medications”, “3–4 Medications” and “5+ Medications”. Ordered logistic regression was applied to examine the effect of time period on the number of medications. Separate models were created for each of the age and gender groups, resulting in six models (model 1: men, 18–45 years; model 2: men, 46–64 years; model 3: men, 65+ years; model 4: women, 18–45 years; model 5: women, 46–64 years; model 6: women, 65+ years). In each model, the outcome or dependent variable was the number of prescribed medications with its four above described categories. The main independent variable was the time period with its three categories: p1 (2005–2007), p2 (2010–2012), and p3 (2015–2017), with p1 being the reference group. Age (as a metric variable, displaying the age of individuals within each age subgroup) and duration of observation (days of observation or insurance within each time period) were added as covariates to all models to adjust for their influence.

Cluster-robust standard errors were used in all models in order to correct for the possible effects of within cluster variation due to having individuals in more than one period, which can lead to autocorrelation.

Ordered logistic regression provides an odds ratio (OR) indicating the odds of being one category higher in the outcome (category of number of medications) for the examined group (p2 or p3) compared to the control group (p1). Even though time period is treated as a predictor variable in the regression models, the aim is to examine the trends of medication prescriptions by interpreting the odds of having a higher number of prescribed medications over time, i.e. in p2 and p3 compared to p1. No additional potential influencing factors were added to the models to concretize the effect of time period within the context of temporal development.

#### 2.4.2. Trend of the Prescription Prevalence

Logistic regression analyses were applied to examine whether there was a significant change in the prevalence of the prescribed medication groups over the three time periods. Cluster-robust standard errors were used to correct for within cluster variation.

In this line of analysis, the outcome or dependent variables were the six medication groups, namely (1) insulin, (2) non-insulin antidiabetic medications, (3) antihypertensive agents, (4) lipid-lowering agents, (5) blood thinning medications, and (6) glycosides and antiarrhythmic medications. Each of these dichotomous outcomes had two categories, yes/no, where “yes” implies having medications prescribed from the corresponding medication group within the examined time period. For each of these outcomes, separate logistic regression models were applied for each gender and age groups, resulting in six models per outcome. Similar to the abovementioned analyses of the trend of the number of prescribed medications, the main independent variable was time period, and age within each age category and duration of observation were adjusted for in all models. 

Since odds ratios tend to either overestimate (if OR > 1) or underestimate (if OR < 1) effects when dealing with outcomes of more than a 10% prevalence rate [18], prevalence ratios (PR) were calculated instead in this analysis.

#### 2.4.3. Predicted Probabilities

Based on the examined regression models described above, predicted probabilities using time period as the main independent variable with margins at means for age and duration of observation were estimated and graphically displayed. Predicted probabilities provide the possibility of interpreting the results more accurately than prevalence rates because they display adjusted effects [19].

The software STATA v15.1 was used for all statistical analyses in this study.

## 3. Results

This study involved 240,241, 295,868, and 308,134 individuals with T2D over the three time periods 2005–2007, 2010–2012, and 2015–2017, respectively. The distributions of age, gender, and insurance durations are presented in Table 1.

### 3.1. Number of Medications

**Figure 1:**
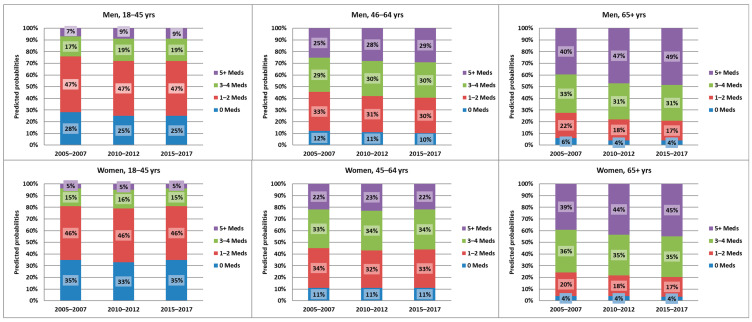
Predicted probabilities of the number of discrete prescribed medications per individual over the three time periods, stratified by gender and three age groups. Meds = Medications.

In men, the number of prescribed medications increased over the three periods among all examined age groups. The predicted probability of having no medications prescribed decreased by up to 3% for the youngest age group, while that of taking five or more medications increased by up to 9% for the oldest age group. Nevertheless, the differences were mostly apparent between the first two time periods, while the change between the periods 2010–2012 and 2015–2017 was minimal (Figure 1).

In women, the change in the number of prescribed medications was only present for the age group of 65+ years. While women in this age group had a slightly lower probability of having only one–two medications prescribed, the probability of having five or more medications prescribed was up to 6% higher during the latest period (Figure 1).

The ordered logistic regression analysis showed that in men, the probability of having at least one more medication prescribed significantly increased over time for all age groups, while it only significantly increased for the age group of 65+ years in women. Men aged 18–45 years were 16% and 21% more likely to have one additional agent prescribed if they were in p2 and p3, respectively (compared to p1). While these probabilities were slightly higher for the middle age group (18% and 22% for p2 and p3, respectively), they were more pronounced for the age group of 65+ years, where men were 30% and 40% more likely to have at least one additional prescribed medication in p2 and p3 respectively. Though significant, the increase was less pronounced for this age group in women, where the odds increased by 21% and 24% for p2 and p3, respectively (Table 2).

### 3.2. Antidiabetic Medications

**Figure 2:**
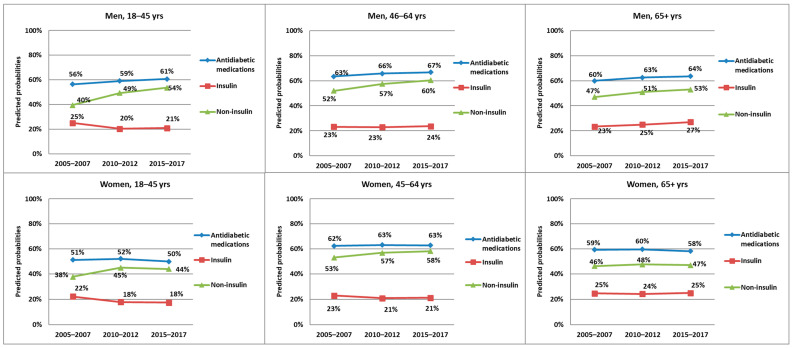
Predicted probabilities of the prescription of antidiabetic medications (at least one of the two subgroups: insulin, non-insulin), insulin, and non-insulin over the three time periods, stratified by gender and three age groups.

In both men and women, the predicted probabilities of having insulin prescribed decreased by 4% for the youngest age group, while the predicted probability of prescriptions entailing non-insulin antidiabetic medications increased considerably, where they were 14% and 6% higher in p3 compared to p1 for men and women, respectively. Though less pronounced, the change in predicted probabilities for insulin and non-insulin medications exhibited similar attitudes for the middle age group. For the oldest age group, however, the predicted probabilities increased for both insulin and non-insulin antidiabetic medications in men but remained almost unchanged in women (Figure 2).

The logistic regression analysis showed that being in the second or the third time periods was significantly associated with a higher chance for non-insulin prescriptions and a lower chance for insulin prescriptions among the youngest and the middle age groups. In the oldest age group, the chances of having both insulin and non-insulin medications prescribed were significantly higher in men, while, in women, only non-insulin prescriptions increased significantly over time (Table 3).

### 3.3. CVD Medications

**Figure 3:**
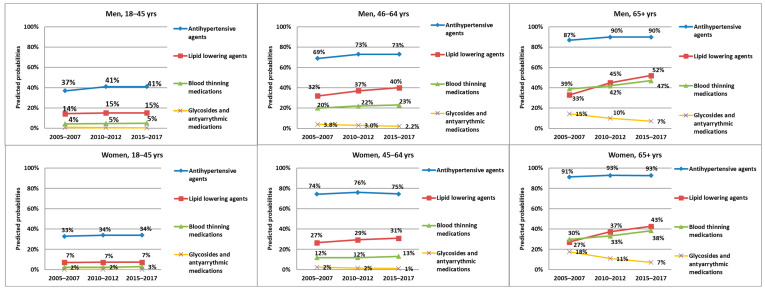
Predicted probabilities of the prescription of CVD medications over the three time periods, stratified by gender and three age groups.

While the prescriptions of antiarrhythmic medications and glycosides were minimal for the two younger age groups in p1, their predicted probabilities slightly decreased over time. For the oldest age group, the predicted probabilities of having prescriptions from this medication group was 15% in men and 18% in women in p1, but these probabilities decreased by more than a half in p3 (Figure 3). These results were also shown in the logistic regression analyses, with significant reductions in the PRs for most of the age and gender subgroups examined (Table 3)

In lipid-lowering and blood thinning medications, there was barely any change in the predicted probabilities for the youngest age group. For the two older age groups, however, there was a clear increase in the predicted probabilities for lipid-lowering and blood thinning medications being prescribed, with the oldest age group being the most affected. While this applied for both genders, men had higher probabilities than women in all three time periods (Figure 3). These conclusions were mostly reproduced through the logistic regression analyses, where it was shown that men and women aged 65 years or older had an up to 38% and 60% higher chance of having lipid-lowering agents prescribed in p2 and p3, respectively. They also had 9–11% and 23–29% higher chances for blood thinning medications in p2 and p3, respectively (Table 3).

The predicted probabilities for antihypertensive agents were the highest among all medication groups examined. Approximately a third of the individuals in the youngest age group had antihypertensive medications prescribed in p1, with an increase by a few percentage points in p2. In the middle age group, 69% of men and three-quarters of women were predicted to have had prescriptions from this medication group in p1. While this probability remained almost constant in women, it increased by a few percentage points in p2 in men. In the oldest age group, there was also a slight increase in the predicted probabilities in p2, but these started off in p1 with 87% and 91% in men and women, respectively. Among all the subgroups, almost no change appeared between p2 and p3 (Figure 3). The logistic regression analyses showed a slight but significant increase in the chance of having this medication group prescribed for all age groups in men and the oldest age group in women (Table 3).

## 4. Discussion

In an attempt to understand possible mechanisms behind different patterns of morbidity expansion in the context of T2D, this study examined the temporal development of medication prescriptions in men and women with T2D.

Overall, the study reported an increase in the number of discrete prescribed medications per individual between the time periods of 2005–2007 and 2015–2017. This is in accordance with the finding from our previous study on the development of comorbidities [7], which reported that the number of comorbidities per individual increased over the same time periods in individuals with T2D. The increase in the number of prescribed medications per person with T2D is also in line with evidence from other European studies. Higgins et al reported a significant increase in the number of prescribed medical agents per person between the years 2000–2015 [10]. Similarly, Oktora et al. reported an increase in “polypharmacy” in individuals with T2D between the years 2012 and 2016 [11]. Nevertheless, while the use of multiple medications can be essential for the treatment of diabetes and its comorbidities, the increase in the number of medications prescribed per person can be associated with related adverse effects and a higher risk for potentially inappropriate medication [11,20].

The trend of the prevalence of antidiabetic medications overall remained constant in women but increased slightly in men. When splitting this group, different attitudes could be observed, pointing towards a decrease in insulin but an increase in non-insulin prescriptions for the two younger age groups. This could be partly attributed to the change in medical therapeutic practices, such as the delay of insulin prescription in individuals with T2D [21,22,23]. A longitudinal study from Germany and UK suggests that the time to insulin therapy as well as the average glycated hemoglobin levels before insulin therapy have increased between 2005 and 2010 [23]. Nevertheless, the increase in the prevalence of non-insulin prescriptions is of a notably higher extent than the decrease in insulin prescriptions in the two younger age groups. In addition, both the prescription prevalence of insulin and non-insulin medications increased for the older age group (65+ years). While this might partly be the result of earlier detection of T2D, it also signposts a deterioration in the management of T2D and an expansion of morbidity in this population, despite changes in prescription practices. Evidence from our previous research which was carried out on the same study population suggests that in the age group of 65+ years, there has been a marked increase in the prevalence of diabetes-related nephropathy [7], for which non-insulin therapy is counter-indicated [24]. Individuals with T2D who suffer from this complication are, thus, left with the only choice of insulin therapy. This in turn also reflects an expansion of the morbidity level, especially among the age group of 65+ years.

Except for glycosides and antiarrhythmic medications that have been prescribed less frequently, possibly due to potential side effects [25], and the existence of medical alternatives, the predicted probabilities as well as the odds for having CVD medications prescribed increased for the two older age groups. Although studies that examined trends for the use of CVD medications in T2D are limited, results from the available evidence from Germany [14,26], as well as other countries, such as Taiwan [27] and the USA [28], designate a similar conclusion based on the trend of CVD medication prescriptions in T2D. Evidence from two German studies indicates that the proportion of individuals with T2D who receive antihypertensive and lipid-lowering medications have increased between 2000 and 2007 [14] and between 1990 and 2011, [26] respectively. The increase in the prevalence of the prescription of CVD medications is consistent with the manifest increase in the prevalence of CVD comorbidities (hypertension, hyperlipidemia, and cardiac insufficiency) that was observed in our previous research carried out on the same population of the current study [7], which reflects a higher morbidity level in this population. Moreover, research also indicates a temporal increase in the prevalence of CVD risk factors in individuals with T2D, such as obesity [29], which can also explain the higher prescription rates of related CVD medications in this population. Nonetheless, changes in medical practices could still be partly responsible for the trend of some CVD medication prescriptions, such as lipid-lowering agents, which had the most pronounced increase among all CVD medication groups between p1 and p3 for both men and women. In 2016, the European Society of Cardiology (ESC) reduced target levels for low-density lipoprotein cholesterol (LDL-C) levels circulating in the blood and, thus, the level from which lipid-lowering medications would be prescribed was decreased [30]. The more recent ESC guidelines from 2019 recommend even lower target blood LDL-C levels [31], which forecasts that apart from the increasing morbidity, a higher prevalence of prescriptions would be potentially observed in future studies that consider later periods.

The increase in the prescription of CVD and antidiabetic medications in T2D could, thus, provide a possible explanation for the different patterns of development of CVD comorbidities in T2D, depending on severity. Our previous findings suggest that the development of severe CVD comorbidities, such as MI and stroke, either remained constant or decreased for some examined subgroups between 2005 and 2017. On the other hand, the predicted probabilities of other comorbidities, such as hypertension and hyperlipidemia that also act as risk factors for MI and stroke, increased markedly and significantly among almost all examined subgroups [7]. It was, thus, hypothesized that differences in medication prescription practices could be associated with the delay of serious health events, such as MI and stroke, in individuals with T2D. The results of the present study support this hypothesis and could be interpreted as an improvement in the medicinal management of risk factors in terms of medication prescription practices, thus, delaying serious health events. The results also reapprove that morbidity is expanding in the population of individuals with T2D. Nevertheless, it still remains an open question whether the expansion of morbidity in T2D in terms of a higher risk of milder CVD comorbidities over time is due to the temporal increase in lifestyle risk factors. Research suggests that lifestyle modification could be more effective than medications in the management of CVD in T2D [32]. The results of a German longitudinal study that examined the development of cardio-metabolic risk factors between the years of 1990 and 2011 suggest that while the prevalence of using antidiabetic, lipid-lowering, and antihypertensive medications increased significantly, there was a simultaneous significant increase in the prevalence of smoking and obesity [26]. Thus, the increase in milder CVD comorbidities that are associated with a deterioration of quality of life in individuals with T2D [29] could be a result of an increase in lifestyle risk factors, such as unhealthy eating habits [33] and lack of adequate physical activity [34], despite the existence of the Disease Management Program (DMP) [35] and a better adherence to the guidelines of the DMP over time [36].

## 5. Strengths and Limitations

The database for this study is routine data of a large population of statutory insured individuals in the state of Lower Saxony, thus, providing adequate power. All medication prescription information is available, ruling out any recall or selection bias. One limitation of the study is that information about the actual intake of medications, and not just prescription information, is not available. However, there is no clear evidence on the existence of temporal differences in the medication adherence in T2D, which makes the three periods comparable in terms of the proportion of individuals who actually took the medications after buying them. Moreover, since the study aims to discuss results in the context of morbidity development, trends of medication prescriptions would presumably reflect how the “need” for these medications have been developing. In addition, certain medication combinations, and not only the number of medications, can be relevant in terms of morbidity development in T2D. However, this was not considered due to the scope of the paper, and will be addressed in future studies. Additionally, the results are not fully generalizable to all individuals with T2D in Germany since the socioeconomic distribution of AOKN differs to some extent from the general population [37].

## 6. Conclusions

This study provides evidence for the temporal increase in the prevalence of medication prescriptions in T2D. The results of this study support the hypothesis of morbidity expansion in the population of T2D. The increase in CVD medication prescriptions, especially lipid-lowering agents, could explain the severity-dependent developmental pattern of T2D comorbidities. Further investigations are planned to examine the temporal development of lifestyle risk factors in T2D to provide a better understanding for the mechanisms behind morbidity expansion in this population.

## Figures and Tables

**Table 1:** Population characteristics.

	2005–2007	2010–2012	2015–2017
N	240,241	295,868	308,134
Age			
18–45 years n (%)	10,893 (4.5)	13,745 (4.7)	14,724 (4.8)
46–64 years n (%)	58,923 (24.5)	80,621 (27.3)	86,585 (28.1)
65+ years n (%)	170,425 (70.9)	201,502 (68.1)	206,825 (67.1)
Gender			
Women n (%)	135,816 (56.5)	159,544 (53.9)	161,222 (52.3)
Men n (%)	104,425 (43.5)	136,324 (46.1)	146,912 (47.7)
Insurance duration in days			
Mean (SD)	1009 (231)	1005 (227)	1003 (242)

**Table 2:** Odds ratios and confidence intervals of the effect of time on the number of medications in individuals with T2D, stratified by age and gender, estimated by means of ordered logistic regression. Adjusted for age (within each age group) and insurance duration.

		18–45 Years n (Men) = 19,340 n (Women) = 20,022		46–64 Years n (Men) = 127,461 n (Women) = 98,668		65+ Years n (Men) = 240,860 n (Women) = 337,892	
		OR	CI	OR	CI	OR	CI
Men	p1	1	-	1	-	1	-
	p2	1.16 *	1.09–1.23	1.18 *	1.16–1.21	1.30 *	1.28–1.32
	p3	1.21 *	1.14–1.29	1.22 *	1.19–1.25	1.41 *	1.39–1.44
Women	p1	1	-	1	-	1	-
	p2	1.07	1.0–1.14	1.08	1.06–1.11	1.21 *	1.19–1.23
	p3	0.99	0.93–1.06	1.04	1.01–1.07	1.24 *	1.22–1.26

* *p* < 0.001.

**Table 3:** Prevalence ratios and confidence intervals on the effect of time period on antidiabetic and CVD medications, stratified by age and gender as estimated by means of logistic regression. Adjusted for age (within each age group) and insurance duration.

			18–45 Years n (Men) = 19,340 n (Women) = 20,022		46–64 Years n (Men) = 127,461 n (Women) = 98,668		65+ Years n (Men) = 240,860 n (Women) = 337,892	
			PR	CI	PR	CI	PR	CI
Antidiabetic medications	Men	p1	1	-	1	-	1	-
		p2	1.05	1.02–1.08	1.04 *	1.03–1.05	1.06 *	1.05–1.07
		p3	1.08 *	1.04–1.11	1.05 *	1.04–1.06	1.05 *	1.04–1.05
	Women	p1	1	-	1	-	1	-
		p2	1.02	0.99–1.05	1.01	1.00.1.02	1.00	0.99–1.01
		p3	0.97	0.94–1.00	1.01	1.00–1.02	0.98 *	0.97–0.98
Insulin	Men	p1	1	-	1	-	1	-
		p2	0.83 *	0.77–0.88	0.99	0.97–1	1.06 *	1.04–1.07
		p3	0.81 *	0.76–0.85	1.02	0.99–1	1.15 *	1.13–1.17
	Women	p1	1	-	1	-	1	-
		p2	0.8 *	0.75–0.85	0.92 *	0.90–0.94	0.98	0.97–0.99
		p3	0.78 *	0.73–0.84	0.93 *	0.90–0.95	1.01	1.00–1.02
Non–insulin	Men	p1	1	-	1	-	1	-
		p2	1.25 *	1.2–1.3	1.09 *	1.08–1.11	1.08 *	1.06–1.09
		p3	1.36 *	1.31–1.41	1.15 *	1.14–1.17	1.11 *	1.10–1.12
	Women	p1	1	-	1	-	1	-
		p2	1.19 *	1.14–1.24	1.07 *	1.06–1.08	1.03 *	1.02–1.04
		p3	1.16 *	1.11–1.21	1.09 *	1.08–1.11	1.02 *	1.01–1.03
CVD Medications	Men	p1	1	-	1	-	1	-
		p2	1.1 *	1.05–1.14	1.05 *	1.04–1.06	1.03 *	1.02–1.03
		p3	1.09 *	1.05–1.14	1.05 *	1.04–1.06	1.04 *	1.03–1.04
	Women	p1	1	-	1	-	1	-
		p2	1.03	0.98–1.08	1.02 *	1.02–1.03	1.01 *	1.01–1.02
		p3	1.03	0.98–1.08	1.01	1.0–1.02	1.01 *	1.01–1.02
Glycosides and antiarrhythmic medications	Men	p1	1	-	1	-	1	-
		p2	0.97	0.64–1.31	0.79 *	0.74–0.84	0.69 *	0.67–0.70
		p3	0.42 *	0.22–0.61	0.58 *	0.54–0.62	0.47 *	0.46–0.48
	Women	p1	1	-	1	-	1	-
		p2	0.69	0.29–1.1	0.66*	0.59–0.72	0.62 *	0.61–0.63
		p3	0.62	0.22–1.03	0.45 *	0.40–0.51	0.41 *	0.40–0.42
Lipid-lowering agents	Men	p1	1	-	1	-	1	-
		p2	1.05	0.96–1.12	1.16 *	1.14–1.18	1.38 *	1.36–1.40
		p3	1.06	0.97–1.14	1.25 *	1.22–1.27	1.6 *	1.58–1.62
	Women	p1	1	-	1	-	1	-
		p2	1.01	0.90–1.12	1.11 *	1.08–1.13	1.37 *	1.36–1.39
		p3	1.01	0.89–1.13	1.17 *	1.14–1.20	1.57 *	1.55–1.59
Antihypertensive agents	Men	p1	1	-	1	-	1	-
		p2	1.11 *	1.07–1.58	1.05 *	1.04–1.06	1.03 *	1.03–1.03
		p3	1.11 *	1.06–1.16	1.05 *	1.04–1.06	1.03 *	1.03–1.04
	Women	p1	1	-	1	-	1	-
		p2	1.04	0.99–1.09	1.02 *	1.02–1.03	1.02 *	1.01–1.02
		p3	1.04	0.98–1.09	1.00	0.99–1.01	1.01 *	1.01–1.02
Blood thinning medications	Men	p1	1	-	1	-	1	-
		p2	1.07	0.93–1.22	1.08 *	1.06–1.11	1.09 *	1.08–1.10
		p3	1.15	0.98–1.31	1.17 *	1.14–1.2	1.23 *	1.22–1.25
	Women	p1	1	-	1	-	1	-
		p2	1.06	0.85–1.28	1.00	0.96–1.04	1.11 *	1.10–1.13
		p3	1.31	1.05–1.56	1.11 *	1.06–1.05	1.29 *	1.27–1.30

* *p* < 0.001.

## Data Availability

The data underlying this study belong to the Allgemeine Ortskrankenkasse Niedersachsen (AOKN-General Local Health Insurance of Lower Saxony). The data are not publicly available due to protection of data privacy of the insured individuals by the AOKN. Interested researchers can send data access requests to Jona Stahmeyer at the AOKN using the following e-mail address: Jona.Stahmeyer@aok.nds.de. The authors did not have any special access privileges.

## Supplementary Materials

The following supporting information can be downloaded at: https://www.mdpi.com/article/10.3390/ijerph20054491/s1, Table S1: ATC codes of medication groups; Table S2: Period prevalence in percent of discrete medication groups in T2D patients by age, sex, and period.

## Abbreviations

AOKN	Allgemeine Ortskrankenkasse Niedersachsen
ATC	Anatomical therapeutic chemical classification
CVD	Cardiovascular diseases
ESC	European Society of Cardiology
LDL-C	Low-density lipoprotein Cholesterol
MI	Myocardial infarction
OR	Odds ratio
p1	Period 1 (2005–2007)
p2	Period 2 (2010–2012)
p3	Period 3 (2015–2017)
PR	Prevalence ratio
T2D	Type 2 diabetes
