# Supplementary material for: Trends of Antidiabetic and Cardiovascular Diseases Medication Prescriptions in Type 2 Diabetes between 2005 and 2017—A German Longitudinal Study Based on Claims Data
Source: Int J Environ Res Public Health. 2023 Mar 3;20(5):4491. doi: 10.3390/ijerph20054491 (PMC10001865; doi:10.3390/ijerph20054491)
Supplement: Supplementary file 1 [file ijerph-20-04491-s001.zip › ijerph-2206893-supplementary.pdf]

Supplementary Materials

Table S1. ATC codes of medication groups.

Medication group	Medication Subgroup	ATC Codes
**Antidiabetic medications**	Insulin	A10A
	Non-insulin	A10B
**CVD medications**	Antihypertensive agents	C01D, C04, C03, C07B, C07C, C07D, C09BA, C09DA. C08G, C07, V07FB, C08, C09BB, C09DB, C09, C02A, C02C
	Lipid-lowering agents	C10
	Blood thinning medications	B01AA, B01AC, B01AF
	Glycosides and antiarrhythmic medications	C01A, C01B

Table S2. Period prevalence in percent of discrete medication groups in T2D patients by age, sex, and period.

		**Males**			**Females**		
**Age**	**Medication**	**p1**	**p2**	**p3**	**p1**	**p2**	**p3**
**18-45 years**	*Insulin*	26.9	21.7	22.9	23.9	19.2	19.0
	Non-insulin	43.3	53.3	57.9	41.5	48.5	47.2
	Blood thinning medications	5.8	6.2	6.2	3.1	3.2	4.0
	Vasodilators	2.1	0.8	0.6	1.4	0.5	0.0
	Diuretics	20.1	20.6	18.1	20.3	19.9	16.0
	Beta blockers	21.6	22.2	19.3	20.3	19.0	16.0
	Calcium channel blockers	8.3	11.5	12.8	6.2	8.4	8.0
	Renin–angiotensin agents	30.6	37.0	37.5	23.4	27.1	27.0
	Lipid-lowering agents	17.3	17.8	17.4	9.5	9.4	9.0
	Glycosides	0.8	0.5	0.2	0.4	0.2	0.0
	Antiarrhythmic medications	0.3	0.5	0.3	0.0	0.1	0.1
	Antiadrenergic agents	2.4	1.8	2.1	2.1	2.5	2.8
**46-64 years**	*Insulin*	24.5	24.3	25.1	24.0	22.1	22.5
	Non-insulin	55.2	60.5	63.9	55.6	59.6	61.2

	Blood thinning medications	21.7	23.6	25.5	12.8	13.0	14.0
	Vasodilators	8.3	5.4	3.1	6.0	3.4	2.0
	Diuretics	43.3	44.2	40.7	51.2	50.1	45.0
	Beta blockers	42.6	44.7	43.0	43.5	44.4	42.0
	Calcium channel blockers	21.1	25.3	26.9	21.3	24.0	24.0
	Renin–angiotensin agents	57.6	64.3	65.9	56.3	62.4	63.0
	Lipid-lowering agents	33.8	39.1	42.1	28.1	31.3	33.0
	Glycosides	3.6	2.4	1.4	2.3	1.4	1.0
	Antiarrhythmic medications	1.2	1.4	1.4	0.6	0.7	0.6
	Antiadrenergic agents	5.7	5.3	5.3	4.8	4.6	4.0
**65+ years**	*Insulin*	26.0	27.5	30.0	27.2	26.9	27.6
	Non-insulin	53.5	56.6	57.9	51.8	52.9	51.9
	Blood thinning medications	41.8	46.8	53.5	32.7	37.0	43.0
	Vasodilators	21.0	15.5	9.2	20.3	13.7	8.0
	Diuretics	67.9	71.9	69.8	79.2	81.0	78.0
	Beta blockers	53.2	62.6	65.3	53.8	64.2	67.0
	Calcium channel blockers	33.4	38.2	39.8	37.5	41.8	43.0
	Renin–angiotensin agents	74.3	82.6	83.9	76.1	82.6	83.0
	Lipid-lowering agents	37.4	50.5	57.9	31.6	42.0	47.0
	Glycosides	14.0	9.2	5.5	18.9	11.5	7.0
	Antiarrhythmic medications	2.6	2.9	3.0	1.5	1.9	2.1
	Antiadrenergic agents	7.6	7.8	7.8	7.6	8.2	8.3
